# Bacterial profile and their antimicrobial susceptibility patterns in patients admitted at MaddaWalabu University Goba Referral Hospital, Ethiopia: a cross sectional study

**DOI:** 10.4314/ahs.v21i2.5

**Published:** 2021-06

**Authors:** Meseret Mitiku Gemechu, Tesfaye Assefa Tadesse, Getahun Negash Takele, Fithamlak Solomon Bisetegn, Yonas Alem Gesese, Tizazu Zenebe Zelelie

**Affiliations:** 1 Department of Medical Laboratory Sciences, MaddaWalabu University, Ethiopia; 2 Department of Medical Laboratory Sciences, Wolaita Sodo University, Ethiopia; 3 Department of Medical Laboratory Sciences, Ambo University, Ethiopia; 4 Department of Medical Laboratory Science, Debre Berhan University, Ethiopia

**Keywords:** Antimicrobial susceptibility patterns, bacterial profile, hospital acquired infections

## Abstract

**Background:**

Hospital acquired infections (HAIs) are one of the global concerns in resource limited settings. The aim of the study was to determine bacteria profile and their antimicrobial susceptibility patterns among patients admitted at surgical and medical wards.

**Methods:**

A hospital based cross-sectional study was conducted from November 2016 to July 2017 in MaddaWalabu University Goba Referral Hospital. Urine and wound swabs were processed and standard disk diffusion test was done to assess susceptibility pattern. Association among variables was determined by Chi-square test.

**Results:**

Among 207 patients enrolled, 24.6% developed HAI, of which, 62.7% and 37.3% were from surgical and medical wards, respectively. The male to female ratio was 1.5:1. The age ranged from 19 to 74 years with a mean of 41.65(±16.48) years. A total 62 bacteria were isolated in which majority of the isolates were gram negative bacteria. Most isolates were resistance to most of the antibiotics tested but sensitive to Ceftriaxone, Norfloxacin and Ciprofloxacin.

**Conclusion:**

Due to the presence of high level drug resistant bacteria, empirical treatment to HAI may not be effective. Therefore, treatment should be based on the result of culture and sensitivity.

## Background

Hospital acquired infections (HAI) are localized or systemic manifestation resulting due to the presence of microorganisms or toxins in the health care settings that was not incubating or symptomatic at the time of admission^1^. It is usually defined as an infection that is identified at least 48–72 hours following admission to health institution^2^. HAI becomes a major global burden posing great threat to patient safety and wellbeing of healthcare providers' audit is associated with increased attributable mortality, length of stay in the hospital, and healthcare costs incurred by patients and healthcare facilities^3^–^6^

Risk factors for such infections vary between different specific site infections. Longer hospital stays, gender, surgery since admission, intubation, mechanical ventilation, age of the patient, type of hospital, urinary catheter were some of the risk factors for hospital acquired infections^7^–^11^. Microorganisms usually implicated in these infections include among others *Pseudomonas aeruginosa, Escherichia coli, Staphylococcus aureus, Klebsiella species*, and *coagulase*-negative *staphylococci*, which are rapidly gaining resistance because of the broad spectrum antibiotics used in an attempt to control them. Most of these organisms are usually contaminants on the surfaces of most materials such as doors, beds, instruments and on care providers. They are therefore easily transmitted to patients when adequate hygienic practices are not followed regularly^12^.

The increased occurrences of HAI related to overuse of antimicrobials, and antimicrobial resistance associated infectious diseases have increased at alarming rate. At present, the emergence of resistance to antimicrobial agents is a global public health problem; particularly in pathogens causing surgical site and urinary infection^13^–^14^. Studies on incidence of HAI in Ethiopia are few and focusing on post-operative HAIs with estimated prevalence of 11% to 36%^15^–^20^. Surgical site and urinary tract infections (UTIs) were found to be commonest forms^17^–^19^. The type of surgery, patients' underlying medical conditions and the type of the ward were found to be important factors associated with increased risk of HAI in Ethiopia^15^–^16^, ^21^–^22^.

The distribution of pathogens causing HAI, especially antimicrobial-resistant pathogens, changes with time and varies across and inside the hospitals. Even though previous studies conducted in the country indicated significant rate of HAI, effective HAI prevention programs are not in place in Ethiopia. In our specific setting, MaddaWalabu University Goba Referral Hospital, there is no such study done. Therefore, the current study was done to determine the bacterial profile and antibiotic susceptibility patterns in patients admitted in surgical and medical wards in MaddaWalabu University Goba referral Hospital, Ethiopia.

## Methods

### Study area

The study was conducted at MaddaWalabu University Goba Referral Hospital, Ethiopia. The Hospital is the largest teaching and referral hospital in Bale Zone, Oromia Regional State, which has 125 beds and is located in the South East of the country, 445 Kms Kilometers from Addis Ababa. It is the only teaching and referral hospital in Bale Zone which provides service for patients coming from different areas of the Zone. In addition to that, many procedures which are risk factors for HAI are performed in this hospital regularly. There was a need of recent and up-to-date information on bacterial profile associated with HAI and their susceptibility patterns which alter over time in the area.

### Study design and study period

A hospital based cross-sectional study was conducted on patients attending surgical and medical wards at MaddaWalabu University, Goba referral hospital from November, 2016 to July, 2017.

### Study population and sample size determination

The study population was patients admitted at medical and surgical wards during the study period and those who fulfill the inclusion criterion. Patients admitted to surgical and medical wards, with greater than 18 years old age and who had suggestive sign and symptoms of UTI or SSI were included in the study. Single proportion formula was used for determining the sample size by considering highest prevalence of healthcare associated infection 16% (P= 0.16)17 in Addis Ababa hospital and 95% level of confidence (α=0.5), with tolerable error of 5% (d=0.05) and Z2 1- α/2 = the standard normal deviation (1.96). The final total sample sized used in the study was 207. HAI is defined as an infection not present or incubating prior to admission to the hospital but generally occurring 48 hours after admission. UTI was defined as positive urine culture (1 or 2 species) having ≥105 bacteria/ml, with or without clinical symptoms. SSI was also defined as any purulent discharge, abscess, or spreading cellulites at the surgical site.

### Sampling techniques and data collection

Patients suspected of HAI were enrolled in the study using convenient sampling technique. Clinical examination was performed by physician to those patients who were admitted to surgical and medical wards. Examination of wounds and catheter entry sites, review of procedures that might lead to infection was made. This was done in order to exclude community acquired infections and to determine any underlying risk factors. A guideline from World Health Organization (WHO) was adopted and it was provided to the physician in order to evaluate those patients. Data on socio-demographic variables (independent variables) include age and sex and associated risk factors include urinary catheter insertion, surgical procedure and duration of admission were collected by structured and pre-tested questionnaire. The outcome variables, bacterial isolates and antimicrobial susceptibility pattern, were recorded in the separate report format.

### Sample collection and transportation

Specimens were collected from patients admitted to the surgical and medical wards and suspected of HAI based on their clinical findings. The specimens were collected using standard operating procedures and analyzed accordingly. Urine sample was collected for bacteriological examination by the mid-stream method (from those who were unable to give mid-stream, catheterization was used) into sterilized container. Urine sample were taken from patients before and after catheterization. Swabs were collected from infected wounds aseptically by using sterile swab. All specimens were transported to the microbiology laboratory for culture and identification. Samples were processed as soon as it reached the laboratory; otherwise, it was placed in the refrigerator at 40°C.

### Identification of bacterial isolates

Urine specimen and wound swab were inoculated on blood agar and MacConkey agar (Oxoid, Ltd, England). The blood agar and MacConkey agar plates were incubated at 37°C for 24–48 hrs. Positive cultures were identified by colony characteristics, Gram-staining reaction and biochemical tests using the standard method^23^. Gram negative bacterial isolates were identified by indole production, H2S production, citrate utilization, motility test, urease test, oxidase test, and carbohydrate utilization. And, Gram-positive bacteria isolates were identified by coagulase, catalase, bacitracin and optochin susceptibility tests.

### Antimicrobial Susceptibility Testing

The antimicrobial susceptibility test was done by the Kirby-Bauer disc diffusion method according to Clinical and Laboratory Standards Institute (CLSI) guideline^24^. Briefly, from a pure culture, 3–5 selected colonies were taken and transferred to a tube containing 5 ml sterile nutrient broth. It was mixed gently until a homogenous suspension is formed. The suspension was incubated at 37°C until the turbidity of the suspension becomes adjusted to a 0.5 McFarland standard. A sterile cotton swab was used and the excess suspension was removed by gentle rotation of the swab against the surface of the tube. The swab was then used to distribute the bacteria evenly over the entire surface of Mueller Hinton agar (Oxoid, England). The inoculated plates were left at room temperature to dry for 3–5 minutes. A sterilized forceps were then being used to lightly press the antibiotic discs manually on the surface of a Muller-Hinton plate to make sure firm attachment. The plates were then incubated at 37°C for 24 hours. Diameters of the zone of inhibition around te discs was measured to the nearest millimeter using a ruler, which was held on the back of the inverted petriplate, and the isolates were classified as sensitive, intermediate and resistant according to the standardized table supplied by the CLSI.

The drugs for disc diffusion test were obtained from Oxoid, England, in the following concentrations:

For Gram negative bacteria; Ampicillin (AMP) (10µg), Amoxicillin-Clavulanic acid (AMC) (30 µg), Chloramphenicol (C) (30µg), Gentamicin (CN) (10µg), Nalidixic acid (NA) (30µg), Nitrofurantin (FM) (300µg), Amoxicillin (AML) (25µg), Tetracycline (TTC) (30µg), Trimethoprim-sulphamethoxazole (TMP-SXT) (25µg), Ceftriaxone (CRO) (30 µg), Doxycycline (DO) (30µg), Norfloxacin (NOR) (10µg) and Ciprofloxacin (CIP) (5µg).

For Gram positive bacteria; Ampicillin (AMP) (10µg), Amoxicillin-Clavulanic acid (AMC) (30µg), Chloramphenicol (C) (30µg), Gentamicin (CN) (10µg), Cloxacillin (CX) (5µg), Methicillin (MET) (5µg), Penicillin G (P) (10 units), Amoxicillin (AML) (25µg), Tetracycline (TTC) (30µg), Trimethoprim-sulphamethoxazole (TMP-SXT) (25µg), Ceftriaxone (CRO) (30µg), Doxycycline (DO) (30µg), Norfloxacin (NOR) (10µg) and Ciprofloxacin (CIP) (5µg). The criterion used to select the antimicrobial drugs is based on guideline provided by CLSI, their availability and from literatures.

### Quality assurance

Standard operating procedures (SOPs) were followed for sample collection, culture, antibiotic and disinfectant susceptibility tests. All antibiotic discs were stored at appropriate temperature. Standard reference strain of *S. aureus* (ATCC-25923), *E. coli* (ATCC-25922) and *P. aeruginosa* (ATCC-27853) were used as a quality control throughout the study for culture and antimicrobial susceptibility testing. After collection, all data including laboratory results were checked for completeness by principal investigator each day and essential feedback was sent to data collectors.

### Data analysis

The socio-demographic, risk factors and microbiological data were collectively documented for each patient on a questionnaire. The information retrieved from these data was analyzed using Statistical Package for Social Sciences (SPSS) version 20. Chi-square test was performed to see the association among dependent and independent variables. A p-value of <0.05 was considered indicative of a statistical significance. Prevalence rate was calculated for the sum of the numbers of positive cases of examined subjects.

## Results

### Characteristics of study participants

In this study, 207 patients suspected for HAI were enrolled for the study. Of the 207 patients, 125 (60.4%) were males and 82 (39.6%) were females with an overall male to female ratio of 1.5:1. The age ranged from 19 to 74 years with a mean of 41.65 (±16.48) years (Table 1). From the surgical site a total of 100 (48.3%) patients and from medical 107 (51.7%) patients were included in the study. The mean hospital stay from the date of admission until sample collection was 9.2 days.

**Table 1:** Age and sex distribution of patients admitted in surgical and medical wards of MaddaWalabu University Goba Referral Hospital from November 2016 to July 2017

Age group	Male, No (%)	Female No (%)	Total
**< 26**	24(11.6)	21(10.1)	45(21.7)
**26–35**	49(23.7)	30(14.5)	79(38.2)
**36–45**	26(12.6)	14(6.8)	40(19.3)
**46–55**	17(8.2)	8(3.9)	25(12.1)
**56–65**	5(2.4)	6(2.9)	11(5.3)
**>65**	4(1.9)	3(1.4)	7(3.4)

**Total**	125(60.4)	82(39.6)	207(100)

### Prevalence of HAI

**Figure 1:**
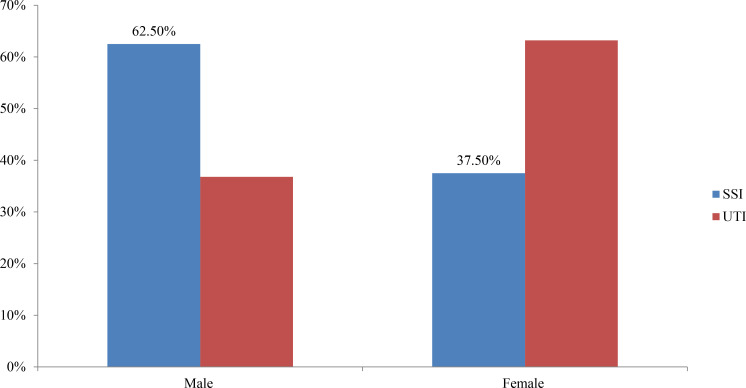
Distribution of Healthcare associated infections by sex among patients (n=51) admitted in surgical and medical wards of Madda-Walabu University Goba Referral Hospital

Among 207 patients, 51 of them were confirmed to have HAI with an overall prevalence of 24.6%. Of which, 30 (58.8%) were males and 21 (41.2%) females. The distribution of HAI among positive cases indicated that 32 (62.7%) were SSI and 19 (37.3%) were UTIs. Of the 32 patients with SSI, 20 (62.5%) were males and 12 (37.5%) were females. Of the 19 patients with UTI, 12(63.2%) were females and 7 (36.8%) were males (Figure 1). The chi-square test indicated that urinary catheterization (p=0.045) and duration of admission (p=0.038) were significantly associated with bacterial HAI. However, age, sex and surgical procedures were not significantly associated with bacterial HAI.

### Bacterial isolates

**Figure 2:**
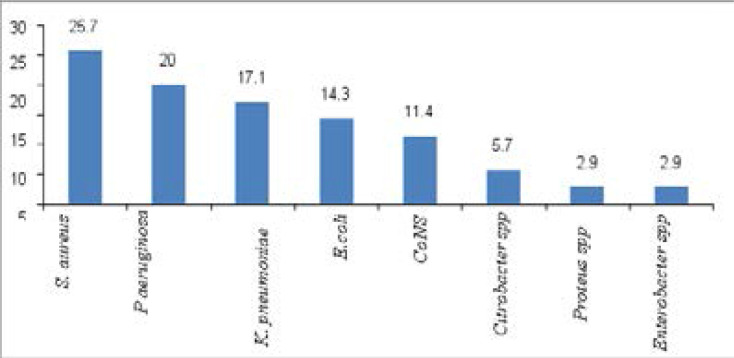
Distribution of bacterial isolates (n=35)from surgical site infected patients admitted in surgical wards of MaddaWalabu university Goba referral hospital from November 2016 to July 2017.

**Figure 3:**
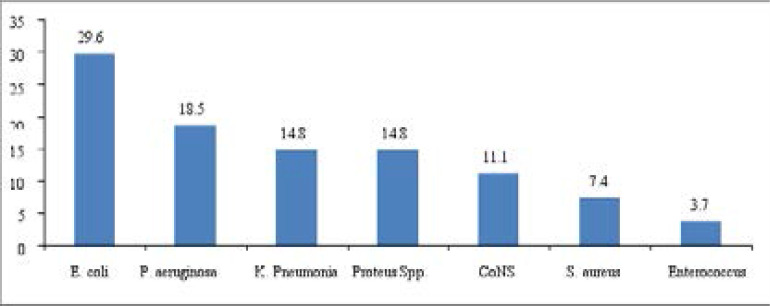
Distribution of bacterial isolates (n=27) from urinary tract infected patients admitted in medical wards of MaddaWalabu University Goba Referral Hospital from November 2016 to July 2017.

A total of 62 bacterial isolates were recovered from HAI (n=51) cases. *E. coli* accounted for 20.9% of the total isolates followed by *P. aeruginosa* (19.4%). More than one bacteria (mixed infection) was isolated from 9/51 (17.6%) of the patients with HAI. The Gram-positive and negative bacteria accounted for 19/62 (30.6%) and 43/62 (69.4%), respectively. Among SSI (n = 35), the predominant isolates was *S. aureus* (25.7%) followed by *P. aeruginosa* (20.0%) and *K. pneumoniae* (17.1%) (Figure 2). Among UTI (n=27), the predominant isolates was *Escherichia coli* (29.6%) followed by *P. aeruginosa* (18.5%) and K. pneumonia (14.8%) (Figure 3).

### Antimicrobial susceptibility pattern of bacterial isolates

#### Gram positive bacteria

All isolates showed high level of resistance (100%) to Ampicillin, Methicillin, Penicillin and Trimethoprim-sulphamethoxazole. On the other hand, most of the isolates were sensitive to Ceftriaxone (63.1%), Norfloxacin (57.9%) and Ciprofloxacin (52.6%) (Table 2).

**Table 2:** Antimicrobial Susceptibility Patterns of Gram-Positive Bacteria (n=19) isolated from patients admitted to surgical and medical wards of MaddaWalabu University Goba Referral Hospital from November 2016 to July 2017

Isolates	Antimicrobial drugs														
		AMP	AMC	CAF	CN	CX	MET	PEN	AML	TTC	SXT	CRO	DO	NOR	CIP
*S.* *aureus* (n=11)	S	-	2	1	-	2	-	-	-	1	-	6	2	4	6
	I	1	-	-	2	-	-	-	-	1	-	-	-	1	-
	R	10	9	10	9	9	11	11	11	9	11	5	9	6	5
CoNS (n=7)	S	-	4	1	2	1	-	-	-	-	-	4	3	4	3
	I	-	-	-	1	-	-	-	1	-	-	2	-	1	-
	R	7	3	6	4	6	7	7	6	7	7	1	4	2	4
Enterococcus *spp.*(n= 1)	S	-	-	-	-	-	-	-	-	1	-	-	1	1	1
	I	-	1	-	-	-	-	-	-	-	-	-	-	-	-
	R	1	-	1	1	1	1	1	1	-	1	1	-	-	-
Total ** (n=19)	S	-	31.6 %	10.5%	10.5%	15.8%	-	-	-	10.5 %	-	52.6 %	31.6%	47.4 %	52.6%
	I	-	5.2%	-	15.8	-	-	-	5.3%	5.3%	-	10.5	-	10.5	-
	R	100	63.2	89	73.7	84.2	100	100	94.7	84.2	100	36.9	68.4	42.1	47.4

S= Sensitive; I=Intermediate; R=Resistant

** Expressed in percent

AMP: Ampicillin; AMC: Amoxicillin-Clavulanic acid; CAF: Chloramphenicol; CN: Gentamicin; CX: Cloxacillin; MET: Methicillin; P: Penicillin; AML: Amoxicillin; TTC: Tetracycline; SXT: Trimethoprim-sulphamethoxazole; CRO: Ceftriaxone; DO: Doxycycline; NOR: Norfloxacin; CIP: Ciprofloxacin

#### Gram negative bacteria

All isolates showed high level of resistance (100%) to Ampicillin, Tetracycline and Trimethoprim-sulphamethoxazole. On the other hand, most of the isolates were sensitive to Norfloxacin (60.5%) (Table 3).

**Table 3:** Antimicrobial Susceptibility Patterns of Gram-Negative Bacteria isolates (n=43) from patients admitted to surgical and medical wards of MaddaWalabu University Goba Referral Hospital from November 2016 to July 2017

Isolates	Antimicrobial drugs													
		AMP	AMC	CAF	CN	NA	NF	AML	TTC	SXT	CRO	DO	NOR	CIP
*E.coli* (n=13)	S	-	3	4	7	5	10	-	-	-	6	3	9	8
	I	-	1	1	2	1	-	1	-	-	1	-	-	-
	R	13	9	8	4	7	3	12	13	13	6	10	4	5
*P.* *aeruginosa* (n=12)	S	-	1	1	1	3	7	-	-	-	1	-	3	2
	I	-	-	1	-	1	1	-	-	-	-	-	2	1
	R	12	11	10	11	8	4	12	12	12	11	12	7	9
*K.* *pneumoniae* (n=10)	S	-	1	2	2	2	5	-	-	-	6	1	5	4
	I	-	-	-	1	2	1	-	-	-	1	1	1	-
	R	10	9	8	7	6	4	10	10	10	3	8	4	6
*Proteus* spp(n=5)	S	-	1	-	3	3	3	-	-	-	2	1	3	2
	I	-	-	-	-	1	-	-	-	-	-	1	1	1
	R	5	4	5	2	1	2	5	5	5	3	3	1	2
*Citrobacter* spp (n=2)	S	-	-	1	-	2	2	-	-	-	1	-	1	1
	I	-	-	-	1	-	-	-	-	-	-	-	-	-
	R	2	2	1	1	-	-	2	2	2	1	2	1	1
*Enterobacter* (n=1)	S	-	-	-	-	-	1	-	-	-	-	-	1	-
	I	-	-	-	1	1	-	-	-	-	-	-	-	1
	R	1	1	1	-	-	-	1	1	1	1	1	-	-
Total (n=43)	S	-	14.0%	18. 6%	30. 2%	34. 9%	65. 1%	-	-	-	37.2%	11. 6%	51.2%	39.5%
	I	-	2.3%	4.7%	11. 6%	13. 9%	4.7%	2.3%	-	-	4.7%	4.7%	9.3%	6.9%
	R	100%	83.7%	76.7%	58.2%	51.2%	30.2%	97.7%	100%	100%	58.1%	83.7%	39.5%	53.6%

S= Sensitive; I=Intermediate; R=Resistant; ** Expressed in percent

AMP: Ampicillin; AMC: Amoxicillin-Clavulanic acid; CAF: Chloramphenicol; CN: Gentamicin; NA: Nalidixic acid; NF: Nitrofurantoin; SXT: Trimethoprim-sulphamethoxazole; CRO: Ceftriaxone; DO: Doxycycline; NOR: Norfloxacin; CIP: Ciprofloxacin

## Discussions

The present study revealed that 24.6% of patients attending surgical and medical wards had bacterial HAI. This is in line with a study conducted in Hyderabad of India 29.13%^25^. However, the finding is higher compared to the studies done in Addis Ababa (13%)^15^ and India (17.6%)^26^, which might be due to poor infection prevention and inadequate service delivery in the study hospital.

In this study, the most prevalent HAI was SSI (62.7%). Since all patients were exposed for surgical procedures, there is an increased risk of getting an infection in the hospital by direct invading of the patient's body, gives bacteria a way in to normal sterile parts of the body. This infection can be acquired from contaminated surgical equipment or from health care workers. In addition, the susceptibility to SSIs were enhanced by poor wound care and prolonged hospitalization.

In the present study, the prevalence of hospital acquired UTIs was 13%. This finding is higher than previous report of study conducted in the country, 6.7%^19^. This may be due to difference in study participants, the present study includes only adults and two wards but the previous study involves all age and wards. In the present study the wards were surgical and medical which have long term admission and catheterization. Catheterization increase the incidence of UTI by 3–5% and long admission increases the probability of acquiring UTI since hospitals are one of the sources of infection. High prevalence of hospital acquired UTIs like 32% in Uganda^27^, and low prevalence 1.4% in Australia^28^ has been reported. The disagreement may be due to difference in infection prevention program implemented in the country. However, the distribution of HAI in the present study agreed with previous studies done in Bahir dar18 and Addis Ababa^15^.

Gram negative bacteria (69.4%) were the dominant isolates than Gram positive (30.6%) in the present study. Similar findings have been observed in Ethiopia^15^, ^16^, and Algeria^29^. This might be due to gram negative bacteria including entrobacteriaceae and non-fermentive gram negative bacilli are significant pathogenic for critical patients. This study found that the most frequent bacterial isolates from SSI were *S. aureus* (25.7%), *P. aeruginosa* (20.0%), and K. pneumonia (17.1%). Likewise, similar findings have been reported from Turkey^30^ and Nigeria^31^. Among UTI bacterial isolates, *E. coli* (29.6%) is the most frequent, followed by *P. aeruginosa* (18.5%), and K. pneumoniae (14.8%). This finding goes in agreement with studies conducted in Ethiopia^32^ and Taiwan^33^.

Nowadays many microorganisms have become resistant to different antimicrobial agents and in some cases to nearly all agents. Resistance to antimicrobial agents is a problem in health care facilities, but in hospitals, transmission of bacteria is amplified because of the highly susceptible population^2^. The antibiotic sensitivity of our study confirmed that an alarming percentage of resistance were exhibited by bacterial isolates to the commonly prescribed antibiotics. In this stance, both Gram-positive and Gram-negative bacteria isolate showed resistance to Ampicillin, Chloramphenicol, Amoxicillin, Tetracycline, and Trimethoprim-sulphamethoxazole. This finding is comparable with studies done in Addis Ababa^34^ and in Gondar^35^.

Gram-negative bacteria showed intermediate level of resistance to Gentamicin. The result has also indicated that Gram-positive were sensitive to Ceftriaxone, Norfloxacin and Ciprofloxacin. Gram-negative bacteria were susceptible to Nitrofurantoin and Nalidixic acid. The Gram positive rate of resistance ranges from 37% to 100% which is comparable with study done in Harar^36^. The Gram negatives rate of resistance also ranges from 40% to 100% which agree with finding reported by Mohammed, et al^35^. Ceftriaxone, Nitrofurantoin, Nalidixic acid, Ciprofloxacin, and Norfloxacin were relatively effective antibiotics for the treatment of pathogens which are responsible to cause HAI. This is perhaps because these agents are not commonly used and newly introduced. However, the uses of drugs like Nalidixic acid, Norfloxacin, and Nitrofurantin are limited in practice because of their higher cost. Due to this fact, they showed low level of resistance. According to this study, it appears that the clinicians are left with very few drug of choice for the treatment of HAI.

## Conclusion

The overall prevalence of bacterial HAI was 24.6%. The dominant HAI was SSI in the present study. Gram negative bacterial isolates, especially Escherichia coli isolates were the predominant in this study. Ceftriaxone, Nitrofurantoin, Nalidixic acid, Ciprofloxacin, and Norfloxacin were relatively effective compared to the other drugs. So, empirical treatment should be based on the result of culture and sensitivity. The capacity of microbiology laboratory should be strengthened with trained manpower, budget and necessary laboratory equipment. There is a need for a continuous surveillance for resistant bacteria to provide the basis of alternative treatment. HAI control should be directed to the hospital and strengthened with different staff, supplies, budget, etc.

## Limitation of the study

The present study has certain limitations. The study does not include all wards in MaddaWalabu University Goba Referral Hospital in which high HAIs are suspected. It was not possible to include anaerobic bacteria due to budget and laboratory facilities constraints. The design of the study did not include fungi and viruses that are important causes of HAI as well. Patients who are able to develop HAI after being discharged were not included in this study. Moreover, the present study were unable to perform molecular tests to confirm the bacterial isolated. So, it is important to conduct molecular test along side with the phenotypic characterization of bacterial isolates.

## Abbrevations

ATCC: American Type Culture Collection; CLSI: Clinical Laboratory Standards Institute; HAI: Hospital Acquired Infection; SOP: Standard Operating Procedure; SPSS: Statistical Package for Social Sciences; SSI: Surgical Site Infection; UTI: Urinary Tract Infection; WHO: World health organization

## Declarations

### Availability of data and materials

All data are already found in the manuscript and there are no supplementary files. The original data supporting this finding will be available at any time upon request of the corresponding author.
